# Rhodacyanine Derivative Selectively Targets Cancer Cells and Overcomes Tamoxifen Resistance

**DOI:** 10.1371/journal.pone.0035566

**Published:** 2012-04-26

**Authors:** John Koren, Yoshinari Miyata, Janine Kiray, John C. O'Leary, Lana Nguyen, Jianping Guo, Laura J. Blair, Xiokai Li, Umesh K. Jinwal, Jin Q. Cheng, Jason E. Gestwicki, Chad A. Dickey

**Affiliations:** 1 Department of Molecular Medicine, USF Health Byrd Alzheimer's Institute, College of Medicine, University of South Florida, Tampa, Florida, United States of America; 2 Departments of Pathology and Biological Chemistry, Life Sciences Institute, University of Michigan, Ann Arbor, Michigan, United States of America; 3 Department of Molecular Oncology, H. Lee Moffitt Cancer Center and Research Institute, Tampa, Florida, United States of America; 4 College of Pharmacy, University of South Florida, Tampa, Florida, United States of America; Mayo Clinic, United States of America

## Abstract

MKT-077, a rhodacyanine dye, was shown to produce cancer specific cell death. However, complications prevented the use of this compound beyond clinical trials. Here we describe YM-1, a derivative of MKT-077. We found that YM-1 was more cytotoxic and localized differently than MKT-077. YM-1 demonstrated this cytotoxicity across multiple cancer cell lines. This toxicity was limited to cancer cell lines; immortalized cell models were unaffected. Brief applications of YM-1 were found to be non-toxic. Brief treatment with YM-1 restored tamoxifen sensitivity to a refractory tamoxifen-resistant MCF7 cell model. This effect is potentially due to altered estrogen receptor alpha phosphorylation, an outcome precipitated by selective reductions in Akt levels (Akt/PKB). Thus, modifications to the rhodocyanine scaffold could potentially be made to improve efficacy and pharmacokinetic properties. Moreover, the impact on tamoxifen sensitivity could be a new utility for this compound family.

## Introduction

MKT-077, a cationic rhodacyanine, has demonstrated cancer specific toxicity and growth inhibition *in vitro* and *in vivo* across multiple cancer varieties [1]. It was determined that MKT-077 localized to the mitochondria [1]. MKT-077 entered into clinical trials for the treatment of advanced and refractory solid tumors of various cellular origin, including: kidney, lung, prostate, colon, adenocarcinomas, and melanomas [2], [3]. The primary negative side effect observed in both studies was renal toxicity [2], [3]. The observed toxicity halted recruitment to one trial as similar animal studies showed irreversible renal toxicity following administration of MKT-077 [2], [3]. Later it was discovered that MKT-077 interacted with mortalin (mot-2), a 70-kda heat shock protein (Hsp70) family member, and that the interaction of MKT-077 with mot-2 induced the release of the tumor suppressor p53 from a complex with mot-2 [4]. This mot-2/p53 complex inactivated the tumor suppression abilities of p53 by sequestering it in the cytosol *in vivo* [5].

Breast cancers are among the most common cancers diagnosed in women [6]. Published data states that treating MCF7 cells, a breast cancer cell model, with MKT-077 produces cytotoxicity and alters growth [1], [2]. However, in the results of two published Phase I clinical trials, no patients with a solid breast tumor or refractory breast tumor were included in the study [2], [3]. Though there are numerous breast cancer chemotherapies, resistance to breast cancer therapies can arise in roughly 30% of women treated for breast cancer [7]. Known resistances in breast cancers have been observed for not only standard anti-cancer strategies, such as doxorubicin, but also trastuzumab and tamoxifen (4-OHT) [8], [9], [10].

Breast cancers also have a high prevalence of mutations; mutations which can promote tumorigenesis and survival [11]. While these mutations produce targets for treatments, other mutations can overcome signaling cascade network circuitry to eliminate upstream targets [12], [13]. This reduces the number of potential targets, reducing the cadre of treatment options, and increasing the potential for resistance genesis. In addition, resistance can emerge when regulatory proteins are altered to allow pro-survival proteins to act unabated. Several kinases related to cell survival have been implicated in facilitating chemotherapy resistance [14], [15], [16], [17], [18]. For example, phosphorylation of the estrogen receptor alpha (ERα) causes ERα to become active regardless of estrogen binding, resulting in resistance to 4-OHT. Thus, strategies to re-sensitize refractory cancer cells to existing therapies are sorely needed.

In these data, we identify a functional derivative of MKT-077 that showed increased cytotoxicity across multiple cancer varieties while still retaining the cancer specificity associated with MKT-077. This enhanced activity was due to the intracellular localization of the compound. In addition, short treatments with YM-1 were able to resensitize cancer cells that had developed resistance to the ERα antagonist, tamoxifen. One way in which these compounds are working is by reducing total Akt levels, which can contribute to ERα insensitivity to tamoxifen. Combined, the rhodacyanine scaffold holds great potential as a cancer therapeutic both as an individual treatment strategy but also, potentially, as a combinational or synergistic option for use with existing regimens.

## Methods

### Cell Lines

Tamoxifen resistant (TR-MCF7) and parental MCF7 cells were generously provided by Dr. Jin Q. Cheng of Moffitt Cancer Center (Tampa, FL). The MCF7 line was originally generated by the Michigan Cancer Foundation and were obtained from ATCC (Manassas, VA) and the TR-resistance was produced by chronic low dose treatment with tamoxifen. HEK-293, M17, H4, MDA-MB-231, Hs578T and NIH-3T3 cells were purchased from ATCC (Manassas, VA). HeLa cells were generously provided by Dr. Kenneth E. Ugen at the University of South Florida. He originally obtained them from ATCC (Manassas, VA). These cells were generated from a cervical tumor from Henrietta Lacks.

### Chemicals and Antibodies

Methylene blue (MB) was purchased from Sigma Aldrich (St. Louis, MO). MKT-077 and YM-1 were synthesized as described [19]. Anti-Akt1, Akt2, and pAktS473 were purchased from Cell Signaling Technology (Danvers, MA). Anti-ERα, and pERα S167 were purchased from Santa Cruz Biotechnology (Santa Cruz, CA). Anti-Actin was purchased from Sigma Aldrich. Anti-GAPDH was purchased from Meridian Life Science (Memphis, TN).

### Cell Culture and Drug Treatments

MCF7, MDA-MB-231, Hs578T and HeLa cells were grown as previously described [20]. H4 and HEK-293 cells were cultured in OPTI - modified Eagle's medium (OPTI-MEM) from Invitrogen supplemented with 10% fetal bovine serum (FBS) and 1% PenStrep (Invitrogen). M17 cells were cultured in OPTI-MEM supplemented with 10% FBS, 1% PenStrep and 100 mg/L Sodium Pyruvate. NIH-3T3 cells were cultured in DMEM with low-sodium bicarbonate (1.5 g/L) from ATCC supplemented with 10% FBS and 1% PenStrep. TR-MCF7 cells were grown in DMEM (described with MCF7 cells) supplemented with 10 µM 4-OHT. MKT-077 and YM-1 were dissolved in DMSO. DMSO was used as vehicle for MKT-077 and YM-1 where indicated. Exact treatment strategies accompany data in results section.

### Protein Collection, Quantitation, and Western Blotting

Cells were harvested by application of mammalian protein extraction reagent (Thermo) as previously described [20]. Protein level measurement, equilibration, western blotting, and detection were performed as previously described [20].

### Lactate Dehydrogenase (LDH) Assay

Indicated cell lines were plated in designated medium. Once cells reached ∼95% confluency, MKT-077 or YM-1 was applied in DMEM without phenol red. After times indicated per experiment, medium was collected from each treatment and centrifuged to pellet dead cells and debris. Protocol was followed as supplied from Cytotox-96 kit (Promega).

### Mitochondrial Isolation and Spectroscopy

MCF7 cells were treated for 6 hours with vehicle (DMSO), MB, YM-1, or MKT-077. Following treatment cells were harvested and subcellular fractions collected using Pierce Mitochondrial Isolation Kit from Thermo Scientific (Rockford, IL). Analysis of drug localization was performed by spectroscopy on Thermo Scientific Nanodrop spectrophotometer. Concentrations and subsequent percentages were approximated by generated concentration:absorbance curve (not shown).

### MTT Cell Viability Assay

TR-MCF7 cells were plated in a 96well plate in medium containing 10 µM 4-OHT. When cells reached ∼90% confluency cells were treated in OPTI-MEM in one of four conditions 1: 10 µM 4-OHT in OPTI-MEM for the full 48 h of experiment. 2: YM-1 (or vehicle) at indicated concentrations for 4 hours followed by exchange of YM-1 medium with medium containing 10 µM 4-OHT. 3: YM-1 (or vehicle) at indicated concentrations for 4 hours followed by exchange of YM-1 medium with medium containing 95% EtOH (vehicle for 4-OHT). Or, 4: YM-1 (or vehicle) at indicated concentrations for the full 48 hours of experiment. MTT assay kit was purchased from ATCC and assay was run as per supplied protocol.

### Isolation of Nuclear Proteins

TR-MCF7 cells were grown in designated medium in 10 cm dishes. Cells were treated for 4 h with 10 µM 4-OHT, 10 µM YM-1, both or vehicle(s) for both compounds. Following incubation, cells were harvested and nuclear proteins isolated using reagents and supplied protocol from the Qproteome Nuclear Protein Kit (Qiagen).

## Results

**Figure 1:**
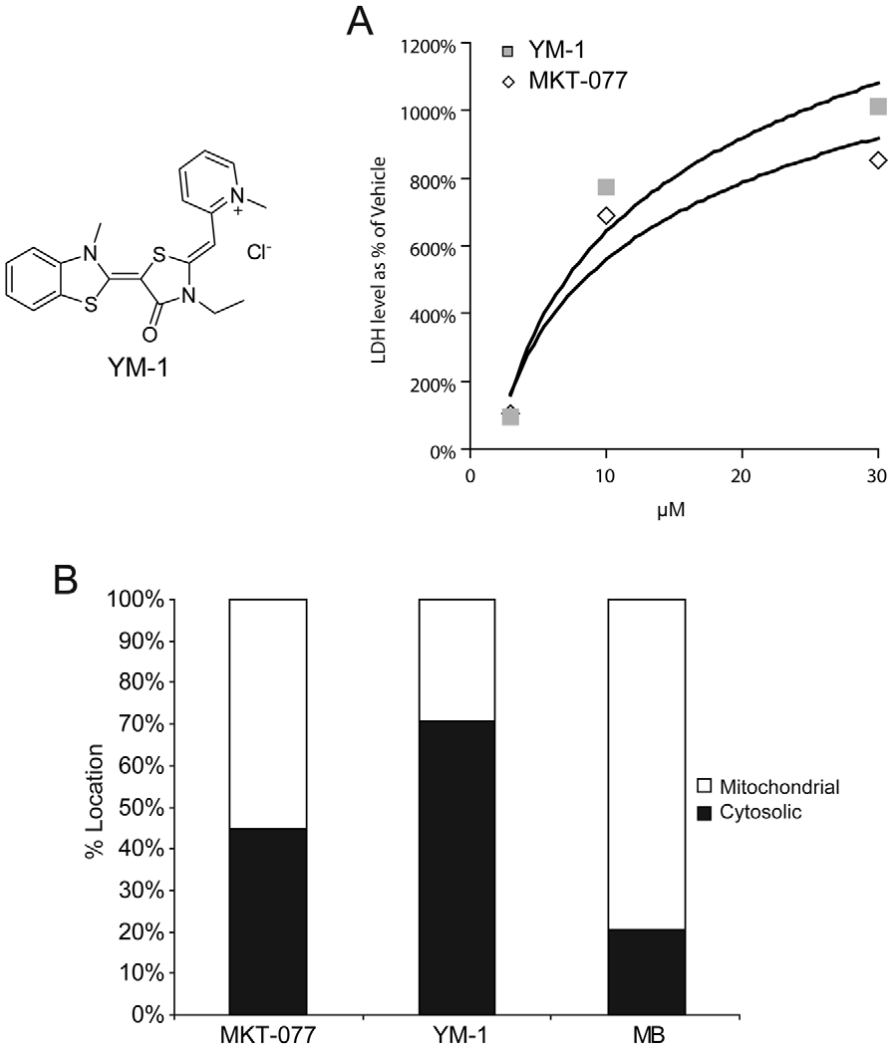
MKT-077 derivative, YM-1, shows enhanced toxicity and altered localization. MCF7 cells were treated for 24 hours with three concentrations of MKT-077 or YM-1. After 24 hours, medium was collected and analyzed by LDH assay. Values shown are a % of vehicle treatment ± SD (A). MCF7 cells were treated with MKT-077, YM-1 or methylene blue (MB). Mitochondrial fractions were collected and compound location was measured by spectrophotometer (B).

The cytotoxicity profiles of a series of derivatives to MKT-077 on MCF7 cells were compared in a small-scale screen. The derivative YM-1 was the only compound found to have dose dependently higher toxicity than MKT-077 after 24 hours (LDH values normalized for cell number)(**Figure 1A**). One possible reason for this improved potency was cellular localization. Taking advantage of the unique spectral properties of these compounds, MCF7 cells were treated with MKT-077 or YM-1 and cellular separation of mitochondria and cytosol were performed. Methylene blue, a compound known to localize to the mitochondria, was used as a control [21]. The subcellular fractions were analyzed spectrophotometrically. These values were compared with a generated standard curve of Abs:concentration (data not shown) to give an approximate concentration of compound in each fraction and thus a percentage of drug per location. Interestingly, YM-1, unlike MKT-077 was more prevalent in the cytosolic fractions (**Figure 1B**).

**Figure 2:**
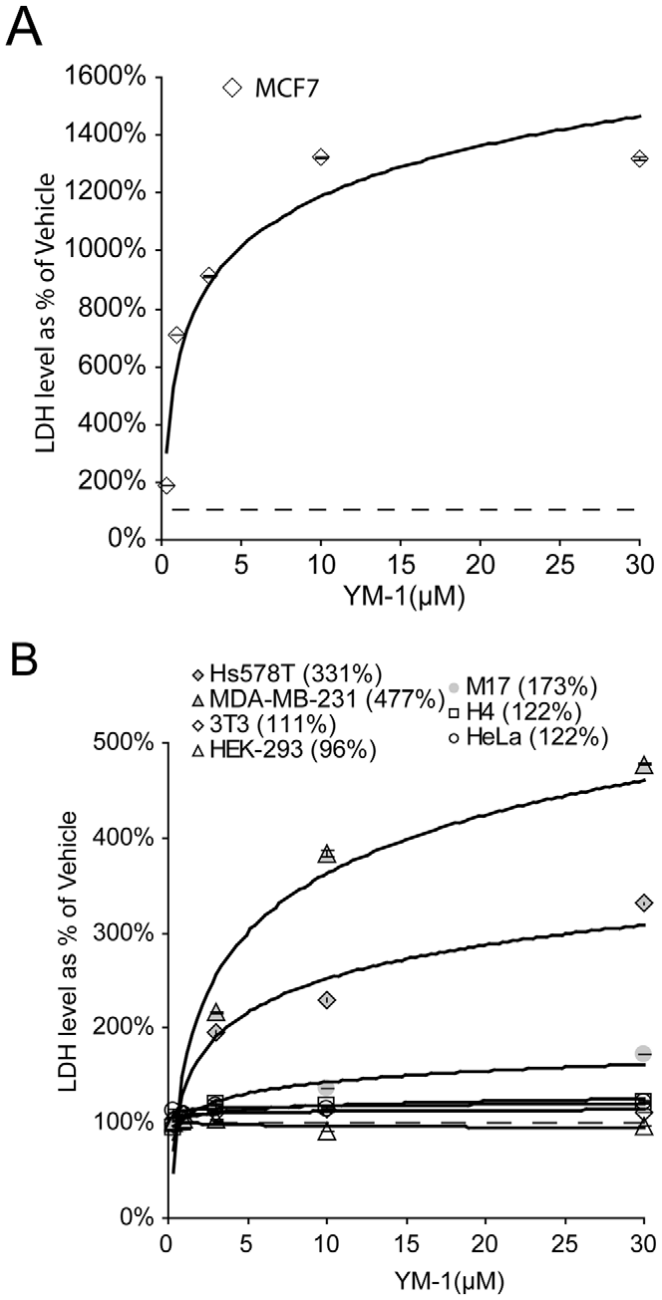
YM-1 toxicity specific to cancer cells; non-cancer cells unaffected. MCF7 cells (breast cancer) were treated for 24 hours with increasing concentrations of YM-1. After 24 hours, medium was collected and analyzed by LDH cytotoxicity assay. Values shown are a % of vehicle treatment ± SD (A). Hs578T and MDA-MB-231 (breast cancer), M17 (neuroblastoma), H4 (neuroglioma), and HeLa (cervical cancer) cell treated with increasing concentrations of YM-1 and the toxicity was compared to NIH-3T3 (mouse embryonic fibroblast) and HEK 293 (human embryonic kidney) cells for cancer specific toxicity. All cell lines were treated for 24 hours. After 24 hours, media were collected and analyzed by LDH assay. Values shown are a % of vehicle treatment ± SD (B).

Concerned that the lack of mitochondrial interaction would reduce the cytotoxic specificity seen with rhodacyanine's for cancer cells, the selectivity of YM-1 on several cancer and immortalized cells lines were tested. These included: MCF7, Hs578T and MDA-MB-231 (breast cancer), M17 (neuroblastoma), H4 (neuroglioma), HeLa (cervical cancer), and two immortalized cell lines: HEK 293 (human embryonic kidney) and NIH 3T3 (murine fibroblast). Robust cytotoxicity (1323% of vehicle), as measured by lactate dehydrogenase (LDH) assay, was observed in MCF7 cells following 24-hour YM-1 treatment (**Figure 2A**). As expected, these toxicity values were higher than those observed in **Figure 1A** since we used a larger cell population (**1A**≈0.2×10^6^ cells; **2A**≈1.2×10^6^ cells) and thus more LDH was released by the associated toxicity. The other cancer cell lines tested all displayed toxicity following YM-1 administration; whereas, the two immortalized cell lines displayed minimal to no toxicity by LDH assay (**Figure 2B**). This demonstrated that the cytosolic localization of YM-1 did not affect its specificity for cancer cells.

**Figure 3:**
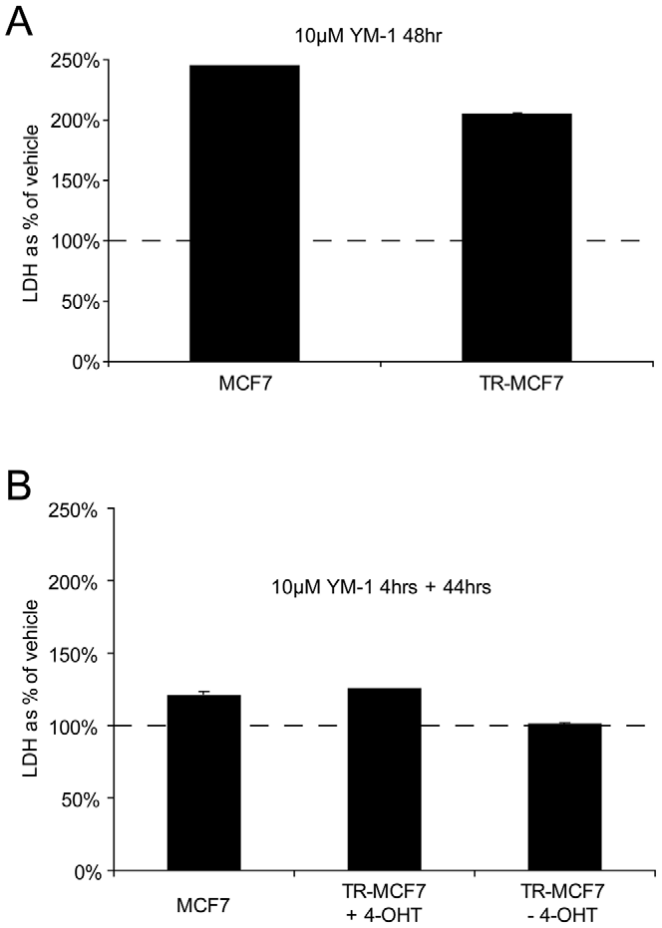
TR-MCF7 cells and MCF7 cells susceptible to YM-1 toxicity at 48 hours but not at 4 hours; tamoxifen does not alter cytotoxicity. TR-MCF7 and parental MCF7 cells were treated for 48 hours in with 10 µM YM-1. After 48 hours, media were collected and analyzed by LDH assay. Values shown are a % of vehicle treatment ± SD (A). MCF7 cells were treated for 4 hours with 10 µM YM-1. At 4 hours, medium was replaced with standard growth media for 44 hours. TR-MCF7 cells were treated with 10 µM YM-1 for 4 hours. At 4 hours, the media was removed and replaced with standard TR-MCF7 media containing 10 µM 4-OHT or 95% EtOH, the vehicle for 4-OHT, for 44 hours. After 48 hours from initial treatment, media were collected and analyzed by LDH assay. Values shown are a % of vehicle treatment ± SD (B).

YM-1 efficacy was then tested in a cell model of tamoxifen-resistance. The toxicity of YM-1 in a refractory tamoxifen (4-OHT) resistant MCF7 (TR-MCF7) cell line was compared to that of the parental MCF7 (non-resistant) cell line. Indeed, YM-1 effectively killed both standard and resistant (TR-MCF7) cells after 48-hour incubation (**Figure 3A**). Given the previous concerns with chronic MKT-077 treatment, we speculated that a shorter treatment with YM-1 might be equally toxic. To test this, MCF7 cells and TR-MCF7 cells were treated with 10 µM YM-1 for 4 hours. This was removed and replaced with vehicle for 44 hours. In addition, TR-MCF7 cells were treated with either 4-OHT or the vehicle for 4-OHT (95% EtOH) (**Figure 3B**). In each case, minimal toxicity was observed.

**Figure 4:**
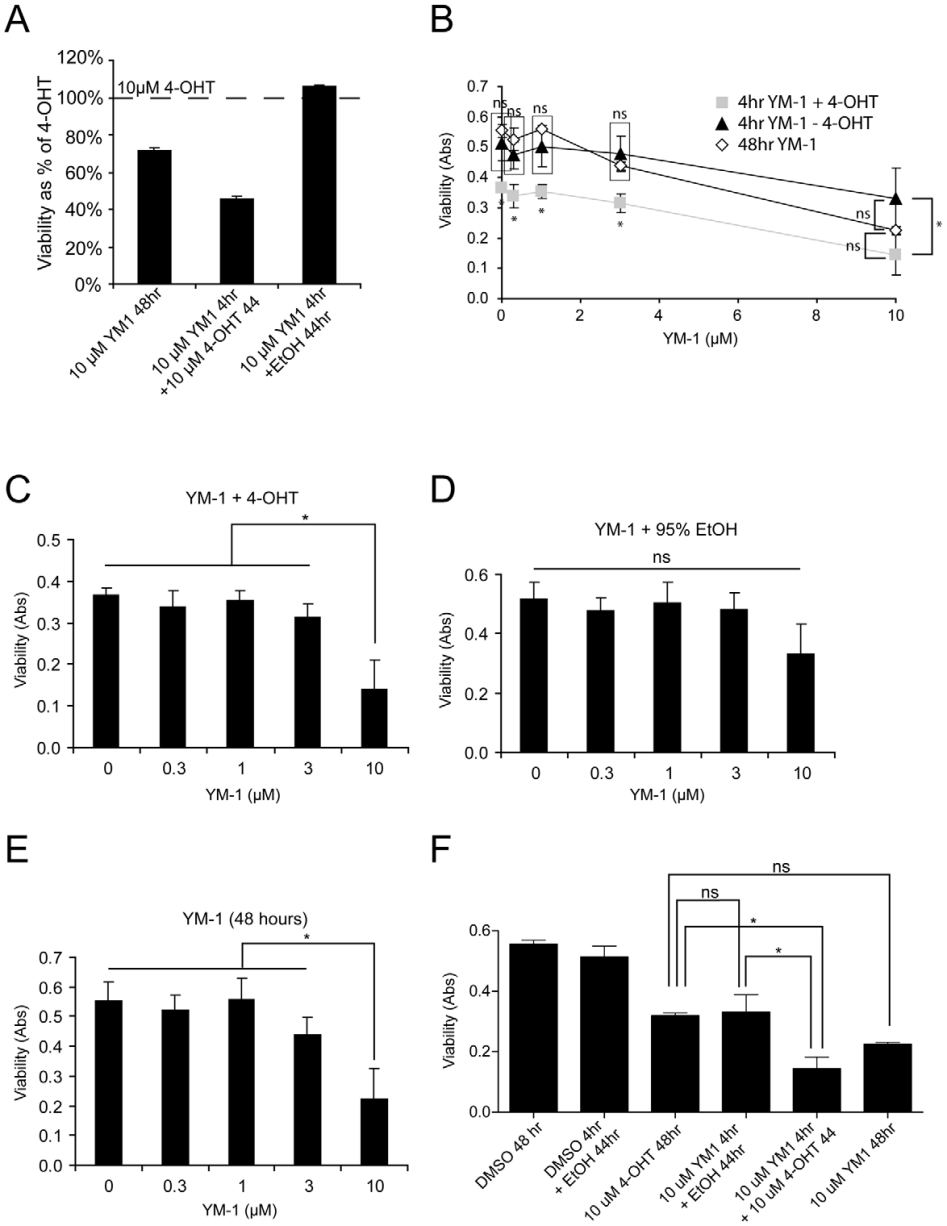
Brief exposure to YM-1 restores tamoxifen effect in resistant cell model. TR-MCF7 cells were treated with 4-OHT for 48 hours, YM-1 (or vehicle) for 4 hours followed by 44 hours of either 4-OHT or 95% EtOH, or YM-1 (or vehicle) for 48 hours. At 48 hours from initial treatment, MTT viability assays were performed. Viability values of each treatment as a % of 48 hours of 4-OHT treatment ± SD (A). 2-Way ANOVA analysis comparing all YM-1 treatment groups (Gray squares- 4-hour YM-1then 4-OHT, open diamonds- 48 hour YM-1, black triangles- 4-hour YM-1 then 95% EtOH), revealed significance across concentrations (F(2,30) = 54.22, p<0.0001), and treatment strategy (F(4, 30) = 41.04, p<0.0001), but no significant interaction (F(8,30) = 1.83, p = 0.1107). * - indicates significant difference (p<0.05) of 4-hour YM-1 then 4-OHT from other two groups with exception of 10 µM treatments, significance as indicated (ns = p>0.05)(B). 1-way ANOVA of YM-1+4-OHT strategy revealing significance of 10 µM concentration (F(4,10) = 16.49, p = 0.0002)(C). Analysis of YM-1+95% EtOH, by 1-way ANOVA, revealed no significance across tested concentrations (F(4,10) = 3.435, p = 0.0516)(D). 48-hour YM-1 treatment showed significance differences between all concentrations and the 10 µM concentration, by 1-way ANOVA (F(4,10) = 12.32, p = 0.0007)(E). 1-way ANOVA analysis (F,(5,12) = 24.33, p<0.0001) comparing all 10 µM YM-1 treatments, 48-hour 4-OHT, and vehicle treatments revealed no significant difference between 48-hour 4-OHT and both 4-hour 10 µM YM-1+95% EtOH and 48-hour 10 µM YM-1 treatments; whereas the 48-hour 4-OHT and the 4-hour 10 µM YM-1+95% EtOH were significantly different from the 4-hour YM-1+4-OHT treatment (p<0.05)(F).

Cell viability (MTT) assays were then used to test whether this shorter treatment strategy was affecting cell proliferation. The TR-MCF7 cells were grown in media containing 10 µM 4-OHT. Our designed treatment strategy contained four conditions all terminating at 48 hours: 1. 10 µM 4-OHT alone for 48 hours, 2. YM-1 (or vehicle) treatment for 4 hours followed by re-addition of 10 µM 4-OHT for 44 hours, 3. YM-1 (or vehicle) treatment for 4 hours followed by 95% EtOH (vehicle for 4-OHT), and 4. YM-1 (or vehicle) treatment for the full 48-hours. MTT assays revealed that the 4-hour 10 µM YM-1 followed by 10 µM 4-OHT treatment reduced viability by 60% relative to the 4-OHT treatment alone. The 10 µM YM-1 followed by 95% EtOH treatment did not alter viability (**Figure 4A**). The 48-hour 10 µM YM-1 treatment reduced viability by 40% compared to 48-hour 4-OHT treatment, similar to **Figure 3A**.

All treatment strategies containing YM-1 were analyzed by two-way ANOVA (**Figure 4B**). This analysis revealed a significant effect by treatment strategy and concentration of YM1 (F(4, 30) = 41.04, p<0.0001), (F(2,30) = 54.22, p<0.0001). The interaction between treatment strategy and concentration was not significant (F(8,30) = 1.83, p = 0.1107). Bonferroni post-hoc analysis of this 2-way ANOVA showed no significant differences between any of the concentrations used for the 48-hour YM-1 treatment and the 4-hour YM-1 followed by 95% EtOH treatment (all p>0.05); whereas, all the concentrations used for the 4-hour YM-1 followed by 4-OHT treatment were significantly different from the 4-hour YM-1 followed by 95% EtOH treatment (all p<0.05). All the concentrations of the 4-hour YM-1 followed by 4-OHT and the 48-hour YM-1 were significantly different (all p<0.05) with the exception of the 10 µM YM-1 concentration (p>0.05). We attributed the lack of significance to the general toxicity caused by the 48-hour 10 µM concentration of YM-1 (see **Figure 3A & B**). A one-way ANOVA of the YM-1 concentration curve for the 4 hour YM-1 treatment followed by 44 hours of 4-OHT treatment revealed that the 10 µM concentration was significantly different from all other concentrations (F(4,10) = 16.49, p = 0.0002)(**Figure 4C**). The concentration curve for the 4 hour YM-1 treatment followed by 95% EtOH treatment displayed that no concentration was significant from any other concentration by one-way ANOVA (F(4,10) = 3.435, p = 0.0516)(**Figure 4D**). Comparison of all of the 48 hour YM-1 concentrations, by one-way ANOVA, displayed that, again, the 10 µM concentration was significantly different from all other concentrations in this treatment (F(4,10) = 12.32, p = 0.0007)(**Figure 4E**). We continued our analysis by comparing the 10 µM YM-1 concentration, from all treatment groups, with the null treatment. Viability values of all aforementioned treatment conditions, with the inclusion of the 4-OHT 48 hour treatment and vehicle treatments as separate groups, were analyzed by one-way ANOVA (F,(5,12) = 24.33, p<0.0001). Tukey's post-hoc test revealed that 4 hour YM-1 followed by 4-OHT was significantly different from the 4-OHT 48 hours treatment (p<0.05), whereas both the 48 hour 10 µM YM-1 and the 4 hour 10 µM YM-1 followed by 95% EtOH treatments were not significantly significant from the 4-OHT 48 hour treatment (**Figure 4F**). This analysis also displayed that 10 µM YM1 followed by 4-OHT is significantly different from 10 µM YM1 followed by 95% EtOH (p<0.05).

**Figure 5:**
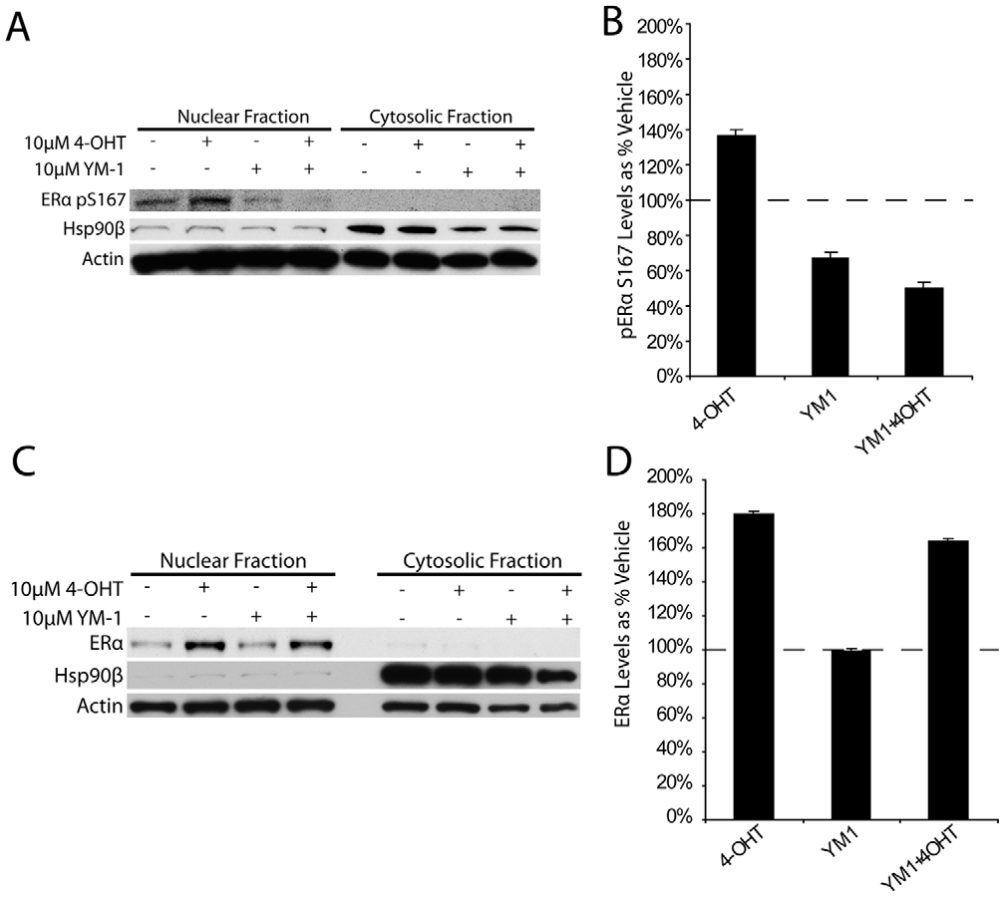
Phosphorylation but not localization of Estrogen Receptor α altered by YM-1. TR-MCF7 cells were treated with indicated conditions for 4 hours. Nuclear isolates and cytosolic fractions were compared by Western blot, representative blots shown (A & C). Densitometry analysis of pERα and ERα levels displayed as % of vehicle treatment ± SD (B & D).

These findings suggested that just a 4 hour treatment of 10 µM YM-1 could re-sensitize TR-MCF7 cells to tamoxifen/4-OHT, stopping cell growth without causing overt toxicity. The potential mechanisms for this phenomenon were then explored. One plausible mechanism was aberrant kinase activity, which is known to promote tamoxifen resistance by phosphorylating ERα at a site known to promote estrogen independent activity [14], [15], [16], [17], [18]. We treated TR-MCF7 cells as described for the experiments in Figure 4A & B. Nuclear proteins were isolated and probed for levels of ERα pS167, a site that when phosphorylated conveys tamoxifen independence. Indeed, phosphorylation of ERα pS167 was elevated in the presence of 4-OHT; however, the addition of 10 µM YM-1 abrogated this event (**Figure 5A & B**). YM-1 did not alter the nuclear localization of ERα into the nucleus (**Figure 5C & D**).

**Figure 6:**
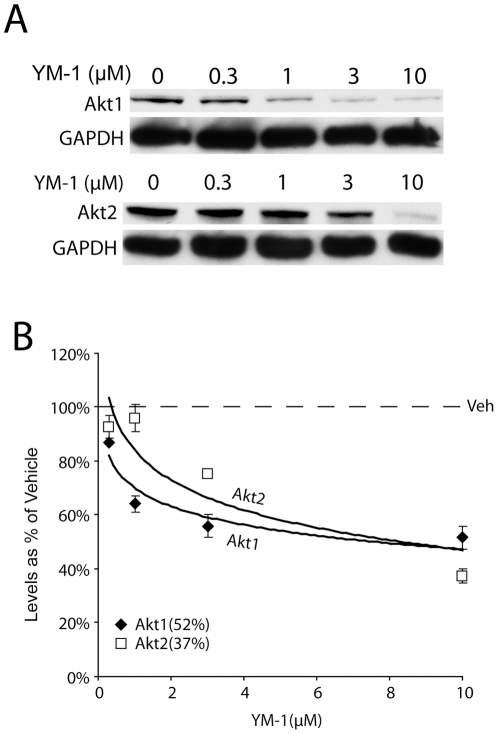
Akt isoforms are concentration dependently reduced by YM-1, potential mechanism for resistance phenomena. MCF7 cells were treated with noted concentrations of YM-1 for 6 hours. Cells lysates were analyzed by Western blot (A). Densitometry analysis of Akt-1 and Akt2 levels shown as % of vehicle treatment ± SD (B).

S167 of ERα falls is contained within an Akt (Akt/PKB) consensus site. Akt is a pro-survival kinase with two isoforms known to interact with ERα. We treated MCF7 cells with 10 µM YM-1 for 6 hours to avoid toxicity and looked for changes in either Akt levels or activation. The levels of Akt1 and Akt2 were dose dependently reduced by YM-1 (**Figure 6A & B**). This suggests that YM-1 can cause toxicity specific to cancer cells, similar to the parent compound MKT-077, but that YM-1 does so by reducing pro-survival kinases like Akt, potentially leading to alterations in resistance mechanisms in refractory tumors. This data agrees with work previous work demonstrating that the effects of LY294002, an inhibitor of the PI3K/Akt signaling pathway inhibitor, on tamoxifen-induced apoptosis were specific for inhibiting Akt activity [16].

## Discussion

Here we describe the therapeutic potential of an MKT-077 derivative, YM-1. This compound, similar to MKT-077, was specifically toxic to cancer cells. YM-1 also had greater efficacy and cytosolic localization than MKT-077. Brief exposure to YM-1 was able to re-sensitize refractory breast cancer cells to tamoxifen, a common therapy used in the clinic. This mechanism was shown to be Akt dependent as YM-1 was able to reduce Akt levels as well as the phosphorylation of ERα at an Akt consensus site. These data demonstrate the potential for rhodacyanine derivatives in the treatment of refractory cancers.

It is possible that the cytosolic presence of YM-1, versus mitochondrial of MKT-077, drives the increased toxicity and Akt clearance observe with YM-1. Though the mitochondrial aspects of MKT-077 are well characterized [1], [2], [3], recent data suggests that MKT-077 can also interact with cytosolic Hsp70 family members [22]. If MKT-077 is able to inhibit cytosolic Hsp70 as it does mortalin [4], [5], YM-1 might also inhibit cytosolic Hsp70. Hsp70 inhibitors have demonstrated cancer specificity as well as the ability to reduce Hsp70 client proteins [20], [23], [24], [25]. We suspect that Hsp70 inhibition could be the mechanism of YM-1; however further examination is required.

Tamoxifen therapies typically fail due to the development of resistance. Acquired resistances take time to develop. Our studies have demonstrated that brief treatments with YM-1 can re-sensitize refractory cancers to tamoxifen. The benefit of such a short treatment is the lack of opportunity for a resistance to YM-1 itself as well as reduced likelihood for off-target toxicities. Moreover, the ability to negate existing resistances allows for the reintroduction of putative chemotherapies; preventing the need for more costly and potentially dangerous secondary and tertiary therapeutic strategies. In addition, YM-1 treatment alone was able to selectively kill only certain cancer cells, suggesting not only tolerability to the approach but also a need for further characterization about the specific cell types that might be sensitive to these compounds and Akt depletion. These benefits to patients coupled with the high number of cancer varieties linked to Akt dysfunction provides a platform for the continued study and development of new compounds to deplete Akt through manipulation of the rhodocyanine scaffold.

While other kinases have been identified to phosphorylated ERα, Akt is a major survival kinase, regardless of resistance phenotype, and its clearance could enhance the efficacy of chemotherapeutics. If our hypothesis that YM-1 is an Hsp70 inhibitor is accurate, this clearance could be due to enhanced ubiquitination of Akt, as the ubiquitin ligase for Akt, CHIP (carboxy terminus of Hsc70 interacting protein) [26], is known to interact with Hsp70 [27].

These studies demonstrate the need for more mechanistic insight into the mode of action of rhodacyanines. Minor changes between MKT-077 and YM-1 lead to an increased cytosolic presence that was able to maintain similar specificity. This suggests that modifications to this scaffold could elicit specific toxicity or reduce renal toxicity, as observed in the clinical and laboratory trials [1], [2], [3]. In fact, our data demonstrating an almost preferential killing of breast cancer cells, versus other cancer cell types, suggests that specificity for particular tumor varieties could be built into the rhodacyanine scaffold.
