# Correction: Rhodacyanine Derivative Selectively Targets Cancer Cells and Overcomes Tamoxifen Resistance

**DOI:** 10.1371/annotation/7493e5d2-4c1a-43eb-a83f-16814861ff13

**Published:** 2012-07-10

**Authors:** John Koren, Yoshinari Miyata, Janine Kiray, John C. O'Leary, Lana Nguyen, Jianping Guo, Laura J. Blair, Xiaokai Li, Umesh K. Jinwal, Jin Q. Cheng, Jason E. Gestwicki, Chad A. Dickey

The eighth author's name was spelled incorrectly. The correct name is: Xiaokai Li. 

